# A Comprehensive Review of TRPS1 as a Diagnostic Immunohistochemical Marker for Primary Breast Carcinoma: Latest Insights and Diagnostic Pitfalls

**DOI:** 10.3390/cancers16213568

**Published:** 2024-10-23

**Authors:** Antonia-Carmen Georgescu, Tiberiu-Augustin Georgescu, Simona-Alina Duca-Barbu, Lucian Gheorghe Pop, Daniela Oana Toader, Nicolae Suciu, Dragos Cretoiu

**Affiliations:** 1 Department of Pathology, “Carol Davila” University of Medicine and Pharmacy, 020021 Bucharest, Romania; antonia.georgescu@umfcd.ro (A.-C.G.); simona-alina.barbu@drd.umfcd.ro (S.-A.D.-B.); 2 Department of Pathology, Clinical Hospital of Nephrology “Dr. Carol Davila”, 010731 Bucharest, Romania; 3 Department of Pathology, National Institute for Mother and Child Health “Alessandrescu-Rusescu”, 020395 Bucharest, Romania; 4 Department of Obstetrics and Gynecology, “Carol Davila” University of Medicine and Pharmacy, 020021 Bucharest, Romania; lucian.pop@umfcd.ro (L.G.P.); oana.toader@umfcd.ro (D.O.T.); nicolae.suciu@umfcd.ro (N.S.); 5 Department of Obstetrics and Gynecology, National Institute for Mother and Child Health “Alessandrescu-Rusescu”, 020395 Bucharest, Romania; 6 Department of Genetics, “Carol Davila” University of Medicine and Pharmacy, 020021 Bucharest, Romania; dragos@cretoiu.ro; 7 Department of Genetics, National Institute for Mother and Child Health “Alessandrescu-Rusescu”, 020395 Bucharest, Romania

**Keywords:** TRPS1, triple-negative breast cancer, immunohistochemistry

## Abstract

Background/Objectives: Immunohistochemical expression of TRPS1 (trichorhinophalangeal syndrome type 1) protein is usually used by pathologists to confirm breast origin for triple-negative breast cancers (TNBC) or metastatic carcinomas of unknown primary. However, recent studies have reported TRPS1 expression in a variety of non-breast lesions. This review aims to provide a comprehensive evaluation of TRPS1 expression across various tumor types, highlighting both its diagnostic utility and potential pitfalls that may arise in clinical practice. Methods: A thorough search of the PubMed database on TRPS1 immunoexpression in tumor pathology was conducted. While the gene itself has been known for several decades, most studies regarding its use in immunohistochemistry emerged in the late 2010s. Particular emphasis was placed on case reports and cohort studies that examined the implications of TRPS1 expression in non-breast tissues, as well as variations in the results between commercially available TRPS1 clones, which may influence the staining intensity and specificity. Results: TRPS1 demonstrated a strong diagnostic utility in identifying primary breast lesions, particularly in TNBC cases. However, its expression in a growing number of non-breast cancers, such as lung adenocarcinoma, prostate adenocarcinoma, urothelial carcinoma, ovarian high-grade serous carcinoma, and endometrial adenocarcinoma, as well as up to 96% of synovial sarcomas with SS18-SSX fusion, emphasizes the need for caution when interpreting TRPS1 positivity and suggests a multi-marker approach in order to increase the diagnostic accuracy. Conclusions: While TRPS1 remains a highly sensible immunohistochemical marker for confirming breast primary lesions, pathologists should be aware of its low specificity and incorporate complementary diagnostic methods in order to ensure accurate clinical management. Further research should focus on elucidating the molecular pathways regulating TRPS1 expression in various tumor types, which may better define its clinical utility.

## Simple Summary

TRPS1 (trichorhinophalangeal syndrome type 1) is a protein that has become an important marker for diagnosing breast cancer, especially in difficult-to-diagnose cases like triple-negative breast cancer. This review explores the expression of TRPS1 in various cancers beyond breast tissue, such as prostate, lung, and ovarian cancers, and discusses its role as a diagnostic tool. While TRPS1 is a highly sensitive marker for breast cancer, its presence in other tumor types poses challenges for pathologists. Our review aims to raise awareness of these diagnostic pitfalls and emphasizes the importance of using multiple markers to improve accuracy. Understanding the broader expression of TRPS1 can help to improve cancer diagnosis and treatment strategies.

## 1. Introduction

The accurate differentiation of primary tumors from metastatic lesions is a fundamental challenge in oncologic pathology. This distinction is particularly critical in breast cancer, a highly prevalent malignancy that often metastasizes to organs such as the lungs, liver, and bones. Immunohistochemistry (IHC) has become an invaluable tool in resolving these diagnostic ambiguities, with an increasing number of antibodies being researched for their utility in differentiating primary breast cancers from metastatic lesions. Among these markers, the zinc finger transcription factor, TRPS1, has recently emerged as a promising candidate [1].

TRPS1 (trichorhinophalangeal syndrome type 1) was initially identified due to its involvement in a rare genetic disorder known as trichorhinophalangeal syndrome, which is characterized by skeletal and facial abnormalities [2]. However, its role in cancer biology, particularly in breast cancer, has garnered significant attention in recent years. TRPS1 is highly expressed in primary breast cancer tissues and has been shown to regulate epithelial–mesenchymal transition (EMT), a process integral to cancer metastasis [3,4].

For pathologists, the consistent expression of TRPS1 in primary breast cancer has made it a useful diagnostic marker, especially in confirming the breast origin for triple-negative breast cancers (TNBCs) and for distinguishing breast cancer metastases from primary malignancies or metastases from other organs. While traditional breast markers such as GATA3, mammaglobin, and estrogen receptor (ER) are commonly employed in diagnostic settings, TRPS1 offers additional sensitivity in complex cases [5,6]. Its immunohistochemical expression pattern in a variety of other tumor types may further expand its utility in the future, but it also currently poses diagnostic challenges for breast pathologists.

This review aims to provide a comprehensive overview of the application of TRPS1 as an IHC marker in breast cancer pathology, focusing on its role in differentiating primary breast cancers from metastatic lesions. Additionally, we will explore TRPS1 expression in other cancers, compare various TRPS1 antibody clones used in clinical practice, and address the pitfalls and challenges associated with its diagnostic application.

## 2. Materials and Methods

The literature review for this article was conducted systematically, following established protocols for scientific reviews. The objective was to gather comprehensive and up-to-date information regarding TRPS1 immunohistochemical expression in primary and metastatic breast cancer, as well as in other tumor types. Relevant studies were identified following a systematic search of a combination of electronic databases, including PubMed, Scopus, Web of Science, and Google Scholar.

The following search terms and Boolean operators were used to maximize the retrieval of relevant studies: “TRPS1 AND breast cancer”, “TRPS1 AND immunohistochemistry”, “TRPS1 AND metastasis”, “TRPS1 AND diagnostic marker”, “TRPS1 AND prostate cancer”, “TRPS1 AND lung cancer”, “TRPS1 AND clone comparison”, and “TRPS1 AND epithelial–mesenchymal transition”. Additionally, combinations of the terms above with “tissue fixation,” “antigen retrieval”, and “immunohistochemical techniques” were employed to identify studies focusing on methodological aspects.

The search covered articles published from 2000 to 2023, encompassing both primary research and review articles. Articles in English were prioritized, though relevant studies in other languages with available English abstracts were also considered.

The inclusion criteria were the following: original research articles, case studies, and clinical trials that focused on TRPS1 expression in human tissues; studies discussing TRPS1 as a diagnostic marker, particularly those comparing primary tumors and metastases; articles exploring the comparison of different TRPS1 antibody clones in IHC; studies that included discussions on TRPS1 expression in non-breast tumors, such as prostate, lung, and gastrointestinal cancers; and studies addressing the role of TRPS1 in epithelial–mesenchymal transition (EMT) and tumor progression.

The exclusion criteria were the following: articles focusing exclusively on the genetic aspects of TRPS1 without discussing its protein expression or IHC application; animal model studies that were not directly applicable to human cancer pathology; abstract-only publications, non-peer-reviewed materials, and editorials without experimental data.

Following the initial search, the retrieved articles were screened for relevance by reviewing the titles and abstracts. Full texts of potentially relevant studies were obtained for a more detailed analysis. The data extracted from each study included tumor type and subtype, TRPS1 expression patterns in both primary tumors and metastases, the diagnostic utility of TRPS1 compared with other immunohistochemical markers, details on the performance and specificity of different TRPS1 antibody clones, as well as information on tissue processing, antigen-retrieval methods, and IHC protocols.

## 3. TRPS1 Immunohistochemistry: Clone Comparison and Performance

TRPS1 has emerged as a significant immunohistochemical marker in the diagnosis of breast carcinoma, particularly due to its sensitivity in distinguishing breast cancer from other malignancies. Several antibody clones have been developed to target TRPS1, including MSVA-512R, EP392, EPR16171, 1D6, 8D11, RM518, OTI3B2, 1B1G2, and 8008R. These clones vary in terms of their staining performance, which is crucial for their clinical utility in diagnosing breast carcinoma, particularly when comparing the sensitivity, specificity, and intensity of staining [7].

In breast carcinoma, TRPS1 typically demonstrates nuclear staining, making it a valuable marker for identifying the breast tissue origin, especially in triple-negative breast cancer (TNBC) and luminal subtypes. For instance, studies comparing TRPS1 antibody clones found that MSVA-512R and 8D11 consistently provide a strong nuclear positivity in the vast majority of breast carcinoma cases. MSVA-512R has shown a high sensitivity, with nearly 100% of invasive breast carcinoma samples staining positive, particularly in luminal subtypes, where intense nuclear staining is often seen. Similarly, clone 8D11 has demonstrated sensitivity rates as high as 92%, though it can exhibit some heterogeneity in staining intensity across different cases of breast carcinoma, especially in TNBC [1,8].

In comparison, EP392 is another clone that has been widely used and validated in breast cancer tissues. This clone shows robust nuclear staining in breast carcinoma, comparable with MSVA-512R, particularly in luminal and HER2-positive subtypes. However, there may be slight differences in the percentage of positive cells and staining intensity when directly compared with MSVA-512R. Notably, TRPS1 and GATA3, when used together, significantly increase the diagnostic accuracy, particularly for TNBC, where traditional markers are often less reliable [9].

The EPR16171 clone also demonstrates a high specificity for breast cancer, with a reliable staining pattern, but has not been as extensively compared with MSVA-512R or 8D11 in the literature. Overall, it seems to perform similarly in breast carcinoma cases with a high sensitivity and specificity [10,11].

When comparing these antibody clones in terms of the staining intensity, studies suggest that MSVA-512R and EP392 provide the most intense and consistent nuclear staining, particularly in luminal and HER2-positive breast carcinoma subtypes. 8D11, while also highly sensitive, may show more variability in intensity, particularly in apocrine differentiation and some TNBC cases. These variations are important when considering the best antibody for diagnostic purposes, as stronger and more consistent staining aids in a clearer interpretation of breast cancer cases [12,13,14].

The successful use of TRPS1 in diagnostic pathology also requires optimized staining protocols, as variations in antigen retrieval, antibody concentration, and detection systems can significantly impact the performance of different clones.

Proper antigen retrieval is essential for maximizing the sensitivity of TRPS1 staining. Heat-induced epitope retrieval (HIER) using a citrate buffer at pH 6.0 has been shown to work well for both the MSVA-512R and EP392 clones. However, some studies have found that a more aggressive retrieval method, using an EDTA buffer at pH 9.0, can improve the sensitivity of TRPS1 staining, particularly in TNBC cases [10].

Optimizing antibody dilution is critical for balancing the sensitivity and specificity. A dilution of 1:100 to 1:200 is typically recommended for FFPE tissue sections, whereas some clones may require a higher concentration (1:50 to 1:100) to produce optimal staining results. The concentration of the antibody should be carefully titrated based on the type of tissue and the staining platform used [1,10].

One of the common challenges with TRPS1 IHC is the potential for cross-reactivity with other zinc finger proteins, particularly those in the GATA family. TRPS1 shares a structural homology with GATA transcription factors, and while its expression is more restricted to breast tissues, there is a risk of cross-reactivity with other cancers, especially when using less specific clones. [12,13,14].

In cases where TRPS1 staining is weak or absent, pathologists must consider several factors, including the possibility of antigen loss due to tissue handling or fixation. Tumor heterogeneity may also lead to variable TRPS1 expression within the same lesion, particularly in metastases, where some areas of the tumor may express TRPS1 while others do not. [5,13].

Furthermore, centralized validation studies involving multi-institutional cohorts would help to assess the reproducibility of TRPS1 staining in different tumor contexts, ensuring that TRPS1 can be reliably used as a diagnostic marker beyond breast carcinoma. This would also allow for better identification of tumor-specific expression patterns and reduce the risk of cross-reactivity, particularly in non-breast malignancies [15].

## 4. TRPS1 as a Diagnostic Immunohistochemical Marker for Primary Breast Carcinoma

In recent years, TRPS1 has gained recognition as a reliable IHC marker in breast cancer pathology due to its consistent overexpression in almost all histopathologic and molecular subtypes of primary breast tumors [5]. This broad expression profile has positioned TRPS1 as a valuable marker for distinguishing breast cancer from other carcinomas that may metastasize to the breast.

Estrogen receptor positivity in luminal-type breast cancers helps pathologists to distinguish those lesions from other malignancies such as gastrointestinal tumors, but triple-negative breast cancers, which, invariably, are hormone-receptor negative, frequently need additional immunohistochemical workup. In such cases, where the histology is not entirely specific to a breast primary lesion, or when dealing with metastases of unknown primary origin, TRPS1 may have important diagnostic relevance. TRPS1 may also be useful in distinguishing luminal-type breast cancers from some gynecological malignancies, where ER/PR expression may pose diagnostic pitfalls [1,13].

One of the key challenges in breast cancer pathology is distinguishing between primary tumors and metastatic lesions, especially in organs that are prone to harboring metastases, such as the liver, lungs, and bone. Metastatic breast cancer often mimics primary tumors in these organs, making histopathological differentiation difficult based on morphology alone. TRPS1 IHC has shown great promise in aiding this differentiation, but, as detailed below, the diagnosis should not rely exclusively on its expression, it should be a corroboration between morphology, other immunohistochemical stains, and the molecular profile [9,13]. Next-generation sequencing (NGS) and other molecular techniques are increasingly utilized to assess genetic alterations that are specific to primary breast tumors. Comparing the mutational profile of a suspected metastatic lesion with the primary breast tumor can confirm the metastatic nature. For instance, somatic mutations in TP53 or PIK3CA, which are frequently seen in breast carcinoma, can support a diagnosis of metastatic breast cancer when found in a distant lesion. Diagnostic approaches should also incorporate the clinical history and imaging findings. Imaging modalities like PET-CT or MRI can help to identify the primary tumor and the metastatic spread pattern, providing critical context. This can be particularly valuable when biopsy samples are limited or inconclusive.

Studies have demonstrated that TRPS1 is highly specific for breast cancer when compared with other carcinomas that are commonly found in metastatic sites [5]. For instance, breast cancer metastases in the lung can histopathologically resemble primary lung adenocarcinomas, but TRPS1 staining is consistently positive in breast cancer and negative in primary lung cancers [9,16]. Similarly, in bone metastases, TRPS1 can help to differentiate metastatic breast cancer from primary bone malignancies, such as osteosarcomas [13]. However, Miao et al. have emphasized the role of decalcification in the immunoreactivity to TRPS1, with the best results observed after decalcification with 10% nitric acid for 6 h.

Furthermore, the specificity of TRPS1 has been shown to outperform traditional markers such as GATA3 in certain diagnostic contexts. While GATA3 is also expressed in a high percentage of breast cancers, its expression can be seen in other tumor types, such as urothelial carcinomas or salivary gland tumors, which may impact the diagnosis [17]. Until recently, TRPS1 has been found to be more specific for breast cancer, reducing the likelihood of misdiagnosis in cases where GATA3 expression might overlap with non-breast malignancies. However, recent publications (discussed below) reveal that the specificity of TRPS1 may not be as high as previously believed.

## 5. Comparison with Other Immunohistochemical Markers for Breast Carcinoma

When dealing with breast cancer metastases, primary breast carcinoma with unusual morphology, or triple-negative breast cancers, TRPS1 is often used in conjunction with other IHC markers to increase the diagnostic confidence. While hormone receptors, mammaglobin, GATA3, and, in some cases, SOX10 remain the primary markers for most subtypes of breast carcinoma, TRPS1 has proven particularly useful in cases where these markers are either absent or equivocal. For example, in TNBC, where ER and PR are not expressed and GATA3 tends to be negative, SOX10 and TRPS1 can provide a much-needed diagnostic anchor [1,5], although both of them may also be positive in other malignant entities.

TRPS1 has shown superior specificity in various clinical settings [18]. For instance, while GATA3 is a highly sensitive marker for breast cancer, its expression in urothelial carcinomas and other tumors, such as parathyroid carcinoma, demands increased caution in its interpretation. TRPS1, on the other hand, is thought to have a more restricted expression pattern, making it a more reliable marker in distinguishing breast cancer from other malignancies [12,13].

Each immunohistochemical marker offers specific benefits and presents its own challenges, particularly in terms of sensitivity, specificity, and utility across different breast cancer subtypes.

### 5.1. Mammaglobin

Mammaglobin is a member of the secretoglobin family and is considered a highly specific marker for breast tissue. It has been widely utilized for confirming both primary breast cancers and metastases from breast carcinoma, particularly in sentinel lymph node biopsies. Several studies have compared mammaglobin with TRPS1, each providing insights into their complementary roles in diagnostics.

**Figure 1:**
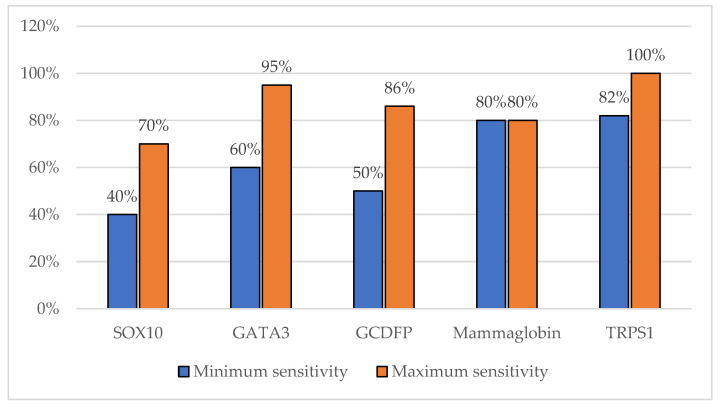
Sensitivity of breast carcinoma markers as reported in the current scientific literature.

Mammaglobin is often regarded as a specific marker for breast tissue, with a high sensitivity for identifying breast carcinoma. Studies have reported mammaglobin expression in up to 80% of breast cancer cases, especially in luminal-type cancers. However, the expression of mammaglobin tends to be more focal and can be weak in poorly differentiated and triple-negative breast cancers (TNBCs), where its sensitivity significantly decreases. In contrast, TRPS1 has been shown to have a higher sensitivity across all molecular subtypes of breast carcinoma, particularly in TNBC, where TRPS1 is more consistently expressed compared with mammaglobin [19]. A brief comparison of the reported sensitivity of breast carcinoma markers can be found in Figure 1.

### 5.2. GCDFP-15 (Gross Cystic Disease Fluid Protien-15)

GCDFP-15 is a well-established immunohistochemical marker that is traditionally used to confirm the breast origin of carcinomas, particularly in metastatic settings. Its expression is most notable in luminal breast carcinomas and apocrine carcinomas, with the reported sensitivity ranging from 50% to 70% across various breast cancer subtypes. However, its sensitivity is significantly lower in triple-negative breast cancers (TNBCs), limiting its utility in these cases. In contrast, TRPS1 has emerged as a more sensitive marker, particularly in TNBC, where it shows a broader expression profile, with sensitivity rates reported as high as 86%. While both markers demonstrate a high specificity for breast tissue, TRPS1 surpasses GCDFP-15 in terms of sensitivity, particularly in poorly differentiated and basal-like breast carcinomas.

### 5.3. GATA3

GATA3 is one of the most widely used markers for breast carcinoma, with a high sensitivity and specificity for breast tissue. It has long been a cornerstone of breast cancer diagnostics, especially in luminal subtypes, and is commonly used in panels alongside ER, PR, and HER2.

GATA3 has a high sensitivity, with expression reported in 90–95% of luminal breast cancers and a high percentage of triple-negative breast cancers (around 60–75%) [5]. However, studies have shown that GATA3 expression can be heterogeneous in TNBC, leading to variable staining, particularly in poorly differentiated tumors. TRPS1, by comparison, has shown more consistent expression across both luminal and triple-negative breast cancers, with a higher sensitivity reported in some studies, particularly in challenging TNBC cases [13]. A study comparing GATA3 and TRPS1 expression in TNBC, revealed that TRPS1 showed higher positivity rates, especially in cases where GATA3 staining was weak or absent [20]. A summary of the comparative studies of the most common immunohistochemical markers for confirming primary breast carcinoma can be found in Table 1.

One of the main diagnostic challenges with GATA3 is its expression in other non-breast malignancies, including urothelial carcinomas, parathyroid tumors, and certain gynecological malignancies. This cross-reactivity can lead to diagnostic confusion, particularly when distinguishing between metastatic breast cancer and a primary malignancy from these other sites [21,22]. It is important to note that immunoreactivity for both TRPS1 and GATA3 has been observed in mesothelioma, warranting caution especially when working up a pleural neoplasm [23,24].

### 5.4. SOX10

SOX10 is a more recent addition to the immunohistochemical panel used in breast cancer, and it is particularly useful in the context of triple-negative breast carcinoma (TNBC). SOX10 is primarily known for its role in melanocytic and neural crest-derived tumors but has gained recognition for its expression in TNBC and basal-like breast carcinomas.

SOX10 has been reported to show expression in approximately 40–70% of TNBC cases, making it a useful marker in identifying basal-like breast carcinomas [25]. However, compared with TRPS1, which demonstrates a more consistent expression across both luminal and TNBC subtypes, SOX10 is more limited in scope. It is especially useful in metaplastic breast carcinomas but shows less reliable sensitivity in other subtypes, particularly in luminal cancers [25,26]. While TRPS1 is expressed in a wider range of breast cancer subtypes, the use of SOX10 is generally limited to specific TNBC cases, often as part of a broader panel including GATA3 and CK5/6.

One of the major limitations of SOX10 is its expression in other non-breast malignancies, including melanoma, salivary gland tumors, and certain nerve sheath tumors [27,28]. This cross-reactivity can lead to diagnostic pitfalls, particularly when metastatic melanoma or a neural crest tumor is part of the differential diagnosis.

SOX10 has found utility in identifying basal-like and metaplastic breast carcinomas, where other markers may not be expressed. However, because of its more limited sensitivity and potential cross-reactivity, it is often used as a supplementary marker rather than a primary diagnostic tool for breast cancer. The broader applicability of TRPS1 across breast cancer subtypes makes it a more robust marker in both routine and challenging cases, particularly in poorly differentiated tumors or TNBC, where SOX10 alone may not provide sufficient diagnostic clarity.

**Table 1:** Summary of comparative studies between the most common immunohistochemical markers for confirming primary breast carcinoma.

Marker	Bradt A et al. [19]	Salem A et al. [29]	Rohra P et al. [30]
TRPS1	31/41 invasive ductal carcinoma 15/15 invasive lobular carcinoma	8/8 mixed cribriform/solid AdCC 14/14 pure-solid AdCC 13/13 TNBC with basaloid features 3/6 cribriform AdCC	55/56 invasive breast cancer
GATA3	40/41 invasive ductal carcinoma 15/15 invasive lobular carcinoma	2/6 cribriform AdCC 2/8 mixed cribriform/solid AdCC 1/14 pure-solid AdCC 3/13 TNBC with basaloid features	37/56 invasive breast cancer

## 6. TRPS1-Negative Breast Carcinoma

**Figure 2:**
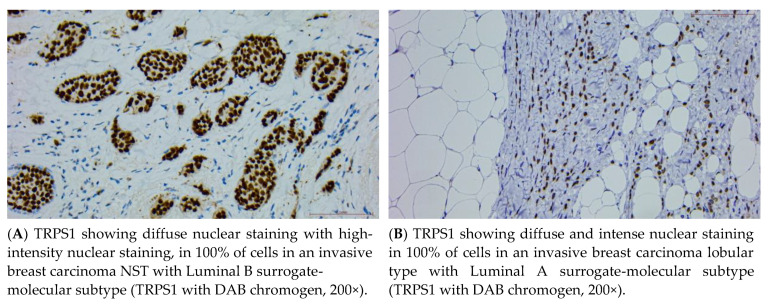

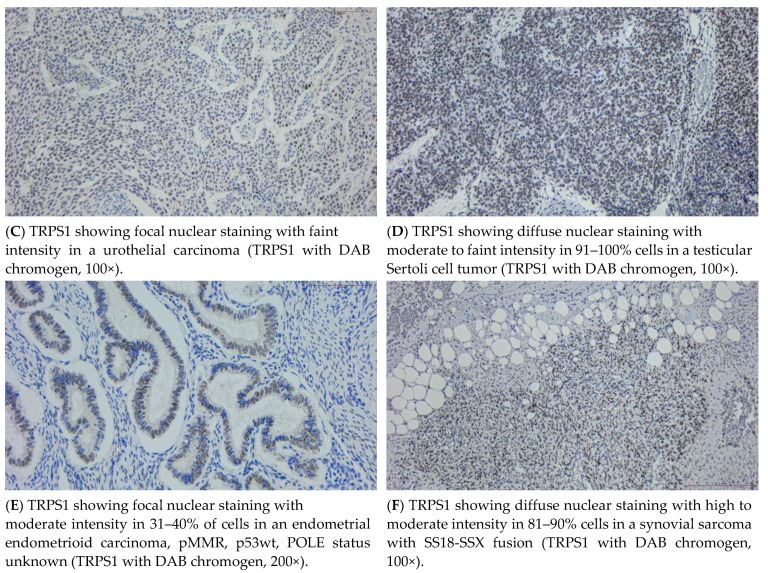
Immunohistochemical micrographs illustrating the different immunoreactivity pattern and staining intensity in different neoplasms.

While TRPS1 is consistently expressed in the majority of breast carcinomas, particularly in luminal and triple-negative subtypes, there are documented cases of TRPS1-negative breast carcinomas. These cases are rare but important to recognize, as they introduce diagnostic challenges, particularly in metastatic settings. Ai et al. has observed that TRPS1 expression is encountered in 98% of ER-positive breast carcinomas and in 58–86% of triple negative breast carcinomas [5,31] (examples in Figure 2A,B). On the other hand, Yoon et al. reported that 100% of triple-negative breast carcinomas express TRPS1 [32].

TRPS1 negativity in breast carcinomas can lead to difficulties in confirming a breast origin, especially when other breast-specific markers, such as GATA3 or ER, are also absent. Several studies have identified subsets of breast carcinomas that lack TRPS1 expression. These TRPS1-negative cases have primarily been reported in apocrine carcinoma, acinic cell carcinoma, cribriform adenoid cystic carcinoma, and neuroendocrine carcinoma of the breast [5,6,33].

Studies that assessed TRPS1 expression across various breast cancer subtypes revealed that approximately 5–10% of TNBC cases are TRPS1-negative [1,34]. Furthermore, basal-like breast carcinomas, which overlap with TNBC, also show variable TRPS1 expression, with a small percentage of cases showing no TRPS1 staining [5]. Kong et al. also observed a statistically significant inverse correlation between the expression of TRPS1 and AR in triple-negative breast carcinoma [5].

Wang et al. reported TRPS1 expression in only 12% of triple-negative breast carcinomas with apocrine differentiation, while GATA3 was positive in 100% of cases. Analogous to mammary apocrine carcinoma, primary cutaneous apocrine carcinoma is also negative for TRPS1 [12]. Additionally, Lennartz et al. observed that 48.6% of Phyllodes tumors and 10% of mucinous carcinomas of the breast were negative for TRPS1 [35]. According to Hu et al., in triple-negative carcinoma, TRPS1 showed the highest rates of expression (53.7%), when compared with SOX10, GATA3, mammaglobin, and GCDFP15 [36]. A panel made out of a novel marker, MGP, used together with GATA3 and TRPS1 can lead to an increased sensitivity in the recognition of all breast carcinomas [13].

The absence of TRPS1 expression in breast carcinomas introduces several potential diagnostic pitfalls. In metastatic cases, where the primary site is uncertain, the lack of TRPS1 expression may lead to difficulty in confirming a breast origin, particularly when other breast-specific markers are also negative. TRPS1 negativity complicates the classification of certain breast cancer subtypes, particularly TNBC and basal-like breast cancers, where the lack of both TRPS1 and other lineage markers makes accurate subtyping challenging. In cases of TRPS1-negative breast carcinomas, over-reliance on TRPS1 alone could lead to a missed diagnosis or misclassification. Pathologists should use a comprehensive panel of markers and consider the clinical context when TRPS1 is negative, especially in poorly differentiated or high-grade tumors.

## 7. TRPS1 Expression in Non-Breast Tumors

### 7.1. Genito-Urinary Tumors

Prostate cancer is one of the most common cancers in men, and its diagnosis often relies on the use of IHC markers such as PSA (prostate-specific antigen) and NKX3.1. However, TRPS1 has been found to be expressed in prostate cancer as well, albeit to a lesser extent than in breast cancer. A recent study by Bachert et al. showed that TRPS1 expression can be observed in up to 31.9% of prostate carcinomas and up to 27.4% of bladder carcinomas [37] (example in Figure 2C). However, only 24.6% of all prostate carcinomas show an intermediate to high expression of TRPS1 [38]. Other studies have shown that an increased expression of TRPS1 is more commonly encountered in patients undergoing androgen withdrawal [38]. Additionally, Van dem Bend et al. demonstrated that LNCaP cells (androgen-responsive cell line that originates from a lymph node metastasis from a prostate carcinoma) that overexpress TRPS1 show a reduction in the expression of androgen-induced PSA in those cells [39]. Other studies have additionally shown that the presence of TRPS1 rs722740 at the TP53 binding site correlates with a more aggressive evolution [40,41]. Lennartz et al. reported the moderate immunoreactivity of TRPS1 in a Sertoli cell tumor of the testis [35].

### 7.2. Pulmonary Carcinoma

Lung carcinoma is a leading cause of cancer-related mortality worldwide, with adenocarcinoma the most common histological subtype. TRPS1 expression has been studied in lung cancer and, interestingly, TRPS1 is typically not expressed in primary lung cancers, making it a useful marker for distinguishing metastatic breast cancer from primary lung adenocarcinoma [31]. However, Chen et al. reported immunoreactivity for TRPS1 in up to 17.4% of lung carcinomas [42]. Additionally, Liu et al. have shown that TRPS1 expression in lung carcinoma is associated with multidrug therapy resistance, through the regulation of the MGMT gene, making this marker a potential future biomarker [43]. On the other hand, Wang et al. showed that 92% of all cases of pleural effusions due to metastatic breast carcinoma will express TRPS1 on the cell block material [9].

However, the absence of TRPS1 in primary lung cancers limits its utility as a standalone diagnostic marker in these cases. Instead, the primary value of TRPS1 in lung cancer diagnostics lies in its ability to exclude metastatic breast cancer in cases where primary lung adenocarcinoma is suspected. This highlights the importance of using TRPS1 in conjunction with other markers, such as TTF-1 and Napsin A, which are specific for primary lung cancers.

### 7.3. Gastrointestinal Cancers

TRPS1 expression has been observed in certain gastrointestinal (GI) cancers, although its diagnostic utility in these contexts is less well-established. Chen et al. reported no immunoreactivity for TRPS1 in colorectal carcinoma [42]. In a study by Hong et al., the expression of TRPS1 in colon carcinoma significantly correlated with the presence of lymph node metastasis and with an advanced pathological stage [44]. However, its expression in colorectal carcinoma is variable and does not appear to be as robust as in breast or prostate cancers.

In gastric carcinoma, TRPS1 expression is similarly heterogeneous. While some studies have reported that TRPS1 expression is seen in up to 45% of cases of gastric carcinoma, others have found little to no expression in these tumor types [5,42]. Additionally, intense TRPS1 staining was observed in the normal gastric mucosa in only 8.3% of cases [45]. The immunoreactivity for TRPS1 was also higher in advanced gastric carcinoma (stage III or stage IV) and in those that featured lymph node metastases [45]. This variability limits the utility of TRPS1 as a diagnostic marker in gastrointestinal malignancies, although it may still hold value in specific cases, particularly when used in conjunction with other markers. Additionally, Lennartz et al. reported TRPS1 reactivity in 22% of GISTs [35].

### 7.4. Gynecological Cancers

Gynecological cancers, including ovarian (Figure 2D), endometrial (Figure 2E), and cervical carcinomas, present unique diagnostic challenges, particularly when they metastasize to organs such as the breast or lungs. TRPS1 expression has been reported in a subset of ovarian and endometrial cancers, although its expression is typically lower than in breast cancer.

In endometrial carcinoma, TRPS1 expression has been reported in a range of between 12.3% to 71% of all tumors [1,42]. Liang et al. also postulated that TRPS1 could be a novel tumor-suppressor candidate, after performing a whole exome sequencing analysis on 13 samples of endometrial carcinoma [46].

In ovarian cancer, TRPS1 expression is seen in up to 20.3% of all ovarian carcinomas [42]. Furthermore, Wang et al. have shown that TRPS1 expression in high-grade serous ovarian cancer correlates with a more aggressive course of evolution of the disease and regulates CCND1 and PSAT1 expression [47]. Additionally, carcinosarcomas of the ovary have been reported to be positive for TRPS1 in 28% of cases [35]. In contrast to Sertoli cell tumors of the testis, Sertoli cell tumors of the ovary were consistently negative for TRPS1 [35].

### 7.5. Synovial Sarcoma

Synovial sarcoma is a rare and aggressive soft tissue malignancy that typically arises near the joints of the extremities, particularly in young adults. The diagnosis of synovial sarcoma can be challenging due to its overlapping histological features with other spindle cell tumors. In recent years, immunohistochemistry has played an important role in aiding the diagnosis of synovial sarcoma, particularly through the identification of the more specific marker, SS18-SSX.

In one study by Cloutier et al., TRPS1 expression was detected in 86% of synovial sarcoma cases, especially in cases associated with SS18-SSX fusion [48]. On the other hand, Giner et al. reported TRPS1 expression in up to 96% of all synovial sarcomas, making it a reliable complementary marker in the diagnosis of synovial sarcoma [49] (example in Figure 2F).

The potential use of TRPS1 in synovial sarcoma diagnosis is still under investigation. In clinical practice, synovial sarcoma is primarily identified using a combination of histological evaluation, molecular testing for SS18-SSX fusion, and IHC markers such as TLE1. Thus, the contribution of TRPS1 to this diagnostic panel could additionally aid in the diagnosis. However, its expression in soft tissue tumors may complicate the use of TRPS1 in distinguishing synovial sarcoma from metastatic carcinomas, particularly when a sarcomatoid carcinoma metastasis is considered in differential diagnoses of spindle cell tumors.

### 7.6. Other Sarcomas

Li et al. reported that the positivity for TRPS1 correlates not only with a lower survival rate in cases of osteosarcoma, but also with the presence of metastases [50]. This expression is probably related to the upregulation of TRPS1 and YAP1 in osteosarcoma cell lines with circTADA2A, enhancing their expression and leading to increased cell proliferation and drug resistance [51]. TRPS1 also plays a role in the development of bone and cartilage tissue, as well as in bone homeostasis deregulation [40,52].

Wang et al. also reported TRPS1 expression in 56% of osteosarcomas and in 11–28% of chondrosarcomas [35,53]. Weak TRPS1 expression has also been observed in rhabdomyosarcoma, tenosynovial giant cell tumor, malignant peripheral nerve sheath tumor, Ewing sarcoma, rhabdoid tumor, and solitary fibrous tumor [35]. TRPS1 expression has also been observed in the sarcomatous component of malignant phyllodes tumors in 95% of cases [54], regardless of if the sarcomatous component was chondro-osseous, liposarcomatous, or spindle cell. Furthermore, 60% of all angiosarcomas of the breast express TRPS1 [14].

### 7.7. Cutaneous Neoplasms

Kim et al. reported TRPS1 positivity in rare cutaneous mesenchymal neoplasms (atypical fibroxanthoma/pleomorphic dermal sarcoma, dermatofibroma, dermatofibrosarcoma protuberans, leiomyoma, and leiomyosarcoma) but also in sarcomatoid squamous cell carcinoma [54,55]. TRPS1 expression in atypical fibroxanthoma and pleomorphic dermal sarcoma might aid, in the future, in establishing this diagnosis, since these tumors are known for not expressing any marker of differentiation [56]. Lennartz et al. also reported reactivity for pilomatricoma in 9.5% of pilomatricomas [35].

Additionally, TRPS1 expression has been reported in up to 94% of squamous cell carcinomas and in 100% of endocrine mucin-producing sweat gland carcinomas, while basal cell carcinomas show no expression or only focal reactivity in 90% of cases [57]. On the opposite side, Lennartz et al. reported reactivity for TRPS1 in only 15% of squamous cell carcinomas of the skin. Furthermore, TRPS1 expression has also been observed in normal eccrine glands and acrosyringia, but not in apocrine glands [35,58].

### 7.8. Salivary Gland Tumors

Among the benign salivary gland tumors, pleomorphic adenoma showed immunoreactivity for TRPS1 in approximately 60–76% of cases and basal cell adenoma showed positivity in 92% of cases [35]. Regarding malignant salivary carcinomas, TRPS1 expression has been reported in 10% of adenoid cystic carcinomas, basal cell adenocarcinomas, and intraductal carcinomas. Lower TRPS1 expression has been observed in polymorphous adenocarcinoma and salivary duct carcinoma [57]. However, according to Salem et al., TRPS1 expression has been observed in only 50% of cribriform adenoid cystic carcinoma [29]. TRPS1 expression has also been observed in adenoid cystic carcinoma of the head and neck and upper respiratory tract, as well as in those of breast origin. These findings suggest that one should not use this marker to differentiate between a salivary gland-type tumor of the breast and one from the head and neck or upper respiratory tract [33].

## 8. Conclusions

TRPS1 has emerged as a valuable immunohistochemical marker in the diagnostic evaluation of breast cancer, particularly in distinguishing primary breast tumors from metastatic lesions. Its expression is robust across various breast cancer subtypes, including luminal, HER2-positive, and triple-negative breast cancers, making it a versatile tool for pathologists. The utility of TRPS1 extends beyond breast cancer, with studies demonstrating its diagnostic potential in a range of other tumor types, including prostate, lung, and gastrointestinal cancers.

However, the use of TRPS1 in diagnostic pathology is not without challenges. Variability in staining protocols, differences between TRPS1 clones, and issues with cross-reactivity highlight the need for the careful optimization and interpretation of the TRPS1 IHC results. Furthermore, tumor heterogeneity and pre-analytical variables can complicate the interpretation of weak or negative staining, underscoring the importance of using TRPS1 in conjunction with other diagnostic markers. The integration of TRPS1 into multi-marker panels, coupled with emerging technologies such as next-generation sequencing and multiplex IHC, holds promise for improving tumor classification and guiding personalized treatment decisions.

TRPS1-negative cases emphasize the importance of a multi-marker approach in breast cancer diagnostics, as relying solely on TRPS1, especially in challenging cases, could lead to diagnostic errors. In these cases, markers such as GATA3, SOX10, mammaglobin, GCDFP15, ER, PR, HER2, and basal markers (e.g., CK5/6) should be included to provide a more complete picture of the tumor’s origin and subtype.

TRPS1 is a valuable addition to the pathologist’s diagnostic toolkit, demonstrating a high sensitivity for breast carcinoma, though with a lower specificity due to its expression in other tumors such as salivary gland tumors, prostate carcinoma, endometrial carcinoma, ovarian carcinoma, gastric carcinoma, synovial sarcoma, and osteosarcoma. The ongoing refinement of TRPS1 IHC and exploration of its broader applications can enhance diagnostic accuracy and support more personalized, effective cancer care.

## Data Availability

The datasets used and analyzed during the current study are available from PubMed, Scopus, Web of Science, and Google Scholar.
